# Clinical Utilization of the FilmArray Meningitis/Encephalitis (ME) Multiplex Polymerase Chain Reaction (PCR) Assay

**DOI:** 10.3389/fneur.2019.00281

**Published:** 2019-03-26

**Authors:** Sara Radmard, Savina Reid, Prajwal Ciryam, Alexandra Boubour, Nhan Ho, Jason Zucker, Dean Sayre, William G. Greendyke, Benjamin A. Miko, Marcus R. Pereira, Susan Whittier, Daniel A. Green, Kiran T. Thakur

**Affiliations:** ^1^Department of Neurology, Columbia University Irving Medical Center, New York, NY, United States; ^2^Department of Infection Prevention and Control, New York Presbyterian Hospital, New York, NY, United States; ^3^Gertrude H. Sergievsky Center, Columbia University Irving Medical Center, New York, NY, United States; ^4^Division of Infectious Diseases, Department of Medicine, Columbia University Irving Medical Center, New York, NY, United States; ^5^Mailman School of Public Health, Columbia University, New York, NY, United States; ^6^Department of Pathology and Cell Biology, Columbia University Irving Medical Center, New York, NY, United States

**Keywords:** meningitis, encephalitis, multiplex PCR, FilmArray, antibiotic stewardship

## Abstract

**Objective:** To assess the clinical utilization and performance of the FilmArray® Meningitis/Encephalitis (ME) multiplex polymerase chain reaction (PCR) panel in a hospital setting.

**Background:** Rapid diagnosis and treatment of central nervous system (CNS) infections are critical to reduce morbidity and mortality. The ME panel is a Food and Drug Administration (FDA) approved rapid multiplex PCR assay that targets 14 bacteria, viruses, and fungi. Previous studies show an overall agreement of 93–99% between the ME panel and conventional diagnostic testing. However, few studies have evaluated the clinical implementation of the ME assay, which is available for routine use at our institution.

**Methods:** We performed a single center retrospective chart review of inpatients who underwent ME panel testing from August 2016 to May 2017. Clinical, radiologic, and laboratory data were reviewed to determine the clinical significance of results. Indication for lumbar puncture (LP), time to results of the ME panel, and duration of antimicrobial therapy were evaluated.

**Results:** Seven hundred and five inpatients underwent ME testing, of whom 480 (68.1%) had clinical suspicion for CNS infection with 416 (59.0%) receiving empiric antimicrobial treatment for CNS infection. The median time-to-result of the ME panel was 1.5 h (IQR, 1.4–1.7). Overall agreement between the ME panel results and clinico-laboratory assessment was 98.2%. Forty-five patients tested positive by ME, of which 12 (26.6%) were determined likely to be clinically insignificant.

**Conclusions:** Routine availability of the ME panel led to overutilization of diagnostic test ordering, as demonstrated by the fact that over one-third of ME panel tests performed were ordered for patients with little or no suspicion for CNS infection. The median time from LP to ME panel result was 1.5 h (IQR, 1.4–1.7). The ME panel's rapid turn-around time contributed to the overuse of the test. Approximately one-quarter of positive ME results were deemed clinically insignificant, though the impact of these positive results requires additional evaluation. Twenty-four and forty-eight hours after the ME panel resulted, 68 and 25% of patients started on empiric therapy remained on antibiotics, respectively. The median time from diagnosis to discontinuation and/or narrowing of antibiotic coverage was 25.6 h (IQR, 3.6–42.5). Further consideration of the appropriate indications for use of the ME panel in clinical settings is required.

## Introduction

Central nervous system (CNS) infections are associated with devastating sequelae, including cognitive deficits, vision and hearing impairment, motor and sensory deficits and epilepsy in over one-half of survivors (1, 2). Rapid diagnosis and treatment of CNS infections are critical to reduce the associated morbidity and mortality. The evaluation of suspected CNS infections is complex, as clinical signs and symptoms are often not specific to the causative pathogen. In many suspected CNS infections, large volumes of cerebrospinal fluid (CSF) are required for diagnostic testing, with long turnaround times for results, which themselves can be affected by prior antimicrobial treatment, timing of LP, and volume of CSF analyzed (3–6). Despite exhaustive efforts to identify an underlying cause, approximately one-fourth to one-half of patients with acute meningoencephalitis remain without an etiologic diagnosis, although it is uncertain what proportion of these cases are infectious or due to other causes (3–11).

Multiplex molecular assays are an attractive option for detection of several microbial targets simultaneously and are now routinely used for bloodstream, respiratory, and gastrointestinal infections (12, 13). In October 2015, the U.S. Food and Drug Administration (FDA) approved the FilmArray Meningitis/Encephalitis (ME) panel (BioFire Diagnostics, Salt Lake City, UT), the first multiplex PCR panel for detection of CNS infections (14). The assay detects 14 pathogens known to cause meningoencephalitis, including six bacteria, seven viruses, and one yeast. The ME panel requires 200 microliters (uL) of CSF with a run time of ~1 h (14).

Since the ME panel was approved, multiple studies have evaluated its performance, yet only a few studies have evaluated clinical implementation. Overall accuracy between gold standard testing and the ME panel is reported between 93 and 99% (15–23). As ME panel testing is increasingly used in clinical practice, more data is needed on the appropriate use of the test and how to interpret results. This study assesses the clinical implementation of routine testing with the ME panel at one hospital setting, evaluating patterns in test ordering, turnaround time, and performance compared to other clinical and laboratory findings.

## Materials and Methods

### Study Design

A retrospective review of patients who underwent diagnostic testing with the ME panel from August 6, 2016 to May 31, 2017 was performed at CUIMC and Children's Hospital of New York (CHONY) (New York, New York). As this retrospective review presented minimal risk to participants, a waiver of consent was granted by the Institutional Review Board of the Columbia University Irving Medical Center. Outpatient cases were excluded due to variations in time of laboratory receipt and limited patient records. This study included patients who underwent lumbar puncture (LP) and had CSF testing with the ME panel. Patients who presented to the emergency department and were either discharged or admitted to the hospital and patients admitted to the pediatric or adult floor, pediatric or adult intensive care unit, or adult bone marrow transplant unit were included in this study. Our study had no age restriction.

### Sample Testing

The ME panel was performed in accordance with the manufacturer's instructions for use. In parallel, all CSF specimens with an ME panel order were required to have concomitant CSF culture orders. In brief, CSF was centrifuged for 10 min at 3,400 rpm and the sediment was vortexed for 30 s to resuspend the pellet. A sterile pipette was used to inoculate the vortexed sediment onto Blood, Chocolate, and MacConkey agar plates, and to prepare Gram stain. The agar plates were cultured aerobically at 35–37° degrees C with 5% CO_2_. Plates were examined at 24 h and re-incubated and examined daily for a total of 5 days. Culture-based identification was performed by MALDI-TOF mass spectrometry (Bruker Biotyper, Bruker Daltonics, Billerica, MA). If ordered, cryptococcal antigen testing was performed by lateral flow immunoassay (CrAg LFA, IMMY Inc., Norman, OK).

Patient electronic medical records (EMR) were reviewed for demographic information, indication for LP, clinical presentation, presence of immunocompromised state, laboratory data including ME panel results, laboratory turnaround time for the ME panel, ancillary testing including neuroimaging results, and antimicrobial initiation and discontinuation times. The initial CSF sample submitted for ME testing was included in our analysis for patients with multiple samples tested during one hospitalization. An abnormal CSF profile was defined as a corrected white blood count ≥5 WBC/mL [correction factor of 750 red blood cells (RBC) to 1 WBC] (24).

Case definitions by organism were based on Infectious Disease Society of America (IDSA) guidelines and included review of clinical presentation, CSF and other ancillary laboratory data, and neuroimaging findings (25). Clinico-radiographic evidence of meningitis included at least two of the following signs: fever, headache, vomiting, nuchal rigidity, and bulging fontanelle as well as CSF pleocytosis (5 or greater white cells/uL) or evidence of meningeal enhancement on brain imaging (26). Major criteria for cases meeting the diagnosis of encephalitis included altered mental status lasting more than 24 h with no alternative source. Minor criteria (at least one for unknown/possible, at least 3 for probable/confirmed) included fever >38°C within 72 h of symptoms or after hospital presentation, generalized or partial seizures not fully attributable to a pre-existing seizure disorder, new onset focal neurological findings, abnormalities of brain parenchyma on neuroimaging, abnormalities on EEG consistent with encephalitis and CSF pleocytosis of 5 or greater white cells/uL (27). Cases meeting criteria for both meningitis and encephalitis were classified as meningoencephalitis and major criteria for the diagnosis of myelitis included sensory, motor, or autonomic dysfunction attributable to the spinal cord and spinal cord lesion on imaging. Minor criteria included fever >38°C and CSF pleocytosis of 5 or greater white cells/uL (26, 27). Two neurologists reviewed the EMR of all patients including physicians' notes, laboratory results, neuroimaging studies and other supporting data to define the significance of ME results. A clinical microbiologist and two transplant infectious disease specialists further reviewed data of those testing positive for HHV-6. To determine the clinical significance and likelihood of positive test results by the ME assay, each reviewer performed an independent probability assessment, assigning cases into one of three categories: likely (>90% probability), possible (10–90% probability), and unlikely (<10% probability). Likely cases presented with one or more clinical signs or symptoms of meningoencephalitis, elevated CSF WBC with or without confirmatory culture or serologic data, and abnormalities on neuroimaging consistent with CNS infection. Possible cases had 2 of 3 above criteria, and unlikely had one or less of the above criteria or a confirmed alternative cause of presentation. For the HHV-6 cases where chromosomal integration interfered with confirmatory testing by IgM/IgG or quantitative PCR in the CSF, patient's clinical presentation, immune status, and neuroimaging were given more weight in determining likelihood of infection. Cases defined as either likely or possible were categorized as clinically concordant cases in our analysis. Cases defined as unlikely were considered clinically discordant cases for the purpose of our analysis. Physicians were blinded to each other's findings. In case of discrepancies, reviewers formally reviewed and discussed their clinical interpretations to arrive at a consensus probability assessment for each case.

### Study Definitions

Time from LP to ME panel result time was defined as time-to-diagnosis. Time from LP to bacterial/fungal culture result for positive cases was designated by the time a pathogen was initially identified whether it was on initial Gram stain or after culture inoculation. Based on CUMC laboratory policy, the culture result time when no pathogen is detected is 5 days for bacteria and 4 weeks for fungi. Empiric antimicrobials were defined as antimicrobials that were initiated prior to organism identification or prior to the ME panel resulting.

### Statistical Methods

The median along with 95% confidence intervals and interquartile range (IQR) were calculated on all continuous variables. The testing result times and time-to-clinical impact data were stratified by age, sex, and race, and compared using two-tailed independent *T*-tests. Comparisons of ME panel and culture turnaround time were performed on using two-sided paired *T*-tests. Further subset analyses were performed on patients who received antimicrobial treatment. A *p* < 0.05 was considered statistically significant in all analyses. All statistical analyses were performed using STATA version 13 software (StataCorp, College Station, TX).

## Results

### Study Population

**Figure 1:**
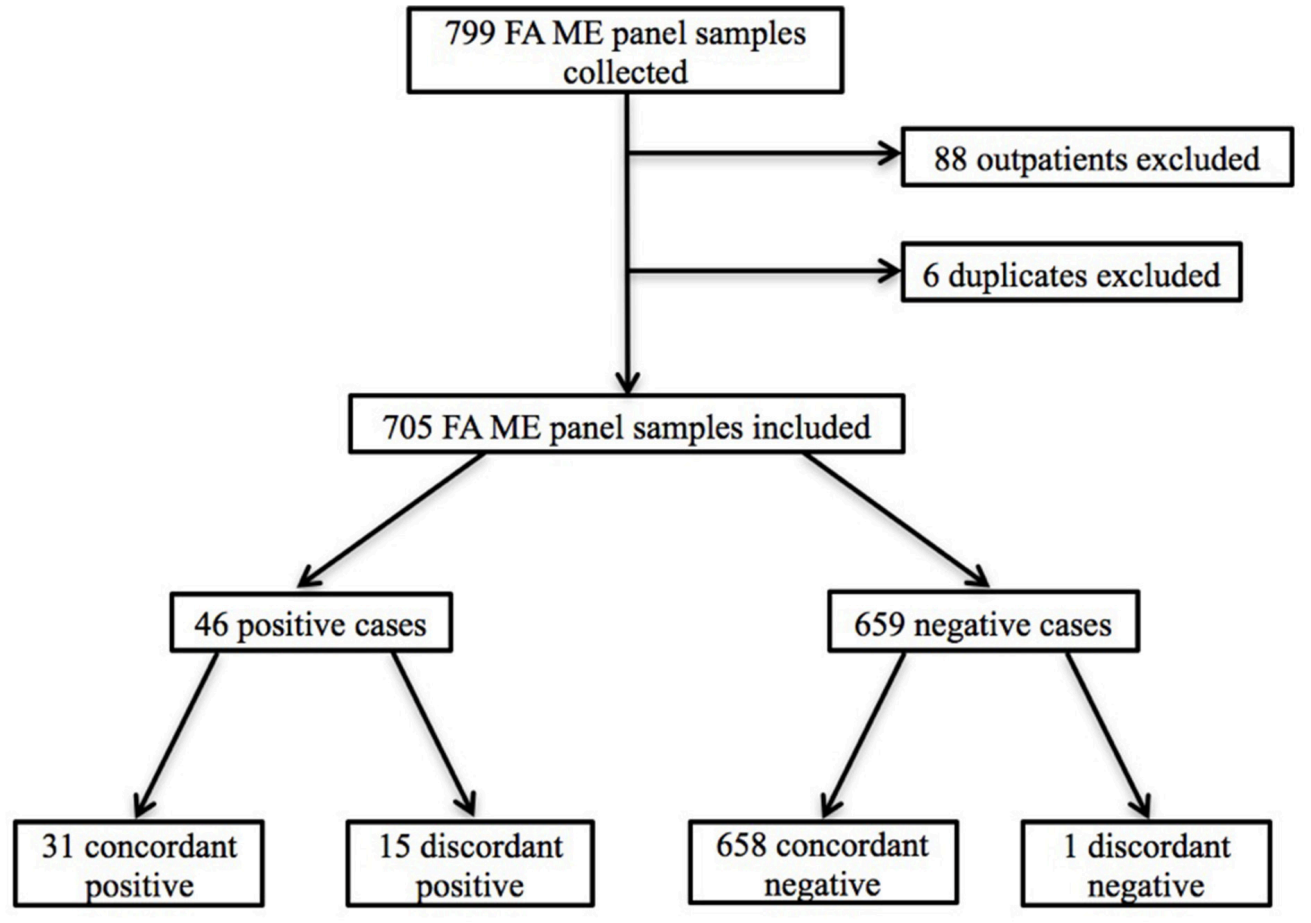
Flowchart of patient inclusion and selection criteria. Outpatients and duplicate ME panels were excluded.

Seven hundred and ninety-nine patients underwent LP with CSF samples sent for ME testing during the study time period. Eighty-eight of these were collected in the outpatient setting and were excluded from our analysis, as were six samples obtained for repeat testing. We analyzed the remaining 705 inpatient CSF samples and corresponding EMRs (Figure 1). Patient ages ranged from 3 days to 95 years with 238 (33.8%) pediatric patients (defined as age younger than 18 years) and 467 (66.2%) adult patients (Table 1). Of those for whom race was documented, 216 (30.6%) were Caucasian, 168 (23.8%) were Hispanic or Latino, 101 (14.3%) were Black/African-American, 18 (2.6%) were Asian/Pacific Islander, and 8 (1.1%) were multiracial patients (Table 1). Four hundred and eighty (68.1%) patients had LP for clinical suspicion for CNS infection and 416 (59.0%) patients received empiric antimicrobial treatment for CNS infection.

**Table 1:** Study patient population demographic data.

	**Study Population (*n* = 705)**
**GENDER**	
Male	363 (51.4%)
Female	342 (48.4%)
**RACE**	
White	216 (30.6%)
African American	101 (14.3%)
Asian/Pacific Islander	18 (2.6%)
Hispanic	168 (23.8%)
Multiracial	8 (1.1%)
Unknown	194 (27.5%)
**AGE**	
Median age	20 (IQR, 0.1–58.0)
Pediatric	238 (33.8%)
Adult	467 (66.2%)
Positive FA cases	33.5 (IQR, 0.8–65.5)
Negative FA cases	18.0 (IQR, 0.1–58)

### CSF ME Panel Results

Of the 705 inpatient samples, there were 45 (6.4%) positive ME panel results. Of the 45 positive ME panel results, the most commonly detected organisms were human herpes virus (HHV)-6 (13 cases, 28.9%), varicella zoster virus (VZV) (7 cases, 15.6%), enterovirus (6 cases, 13.3%), human parechovirus (4 cases, 8.9%), and *Streptococcus pneumoniae* (4 cases, 8.9%). *Haemophilus influenzae, Listeria monocytogenes*, and *Neisseria meningitidis* were not detected during the study period (Table 2). In one patient, the ME panel detected cytomegalovirus (CMV) then *Cryptococcus* 24 days later during another admission. There were no cases where multiple infectious pathogens were detected in one sample.

**Table 2:** Number of pathogens detected and performance outline of FilmArray ME Panel.

**Organism identified**	**FA ME panel detection (n)**	**True positive**	**False positive**	**True negative**	**False negative**	**Sensitivity**	**Specificity (%)**
**BACTERIA**							
*E. Coli K1*	1	1	0	704	0	100%	100
*H. influenzae*	0	0	0	705	0	N/A	100
*L. monocytogenes*	0	0	0	705	0	N/A	100
*N. meningitidis*	0	0	0	705	0	N/A	100
*S. agalactiae*	2	2	0	703	0	100%	100
*S. pneumoniae*	4	3	1	701	0	100%	99.80
**YEAST**							
*C. neoformans/gattii*	1	1	0	704	1	50%	100
	**FA ME panel detection (*****n*****)**	**Concordant positive**	**Discordant positive**	**% Concordance**			
**VIRUSES**							
CMV	2	1	1	50			
EV	6	6	0	100			
HSV-1	2	2	0	100			
HSV-2	3	3	0	100			
HHV-6	13	3	10	23.10			
HPeV	4	4	0	100			
VZV	7	7	0	100			

*Sensitivity and specificity calculations for bacteria and yeast are based on comparison to gold standard culture results. Concordance for viruses is based on chart review to determine the clinical significance of cases*.

Twelve (26.7%) cases testing positive by ME subsequently yielded negative results on confirmatory testing and/or were deemed to be clinically insignificant by clinician review. The sensitivity for the ME panel compared to culture for bacteria and fungi was 100 and 50%, and the specificity was 99.9 and 100%, respectively. Four cases of *S. pneumoniae* were detected, with one case (25.0%) deemed discordant based on negative culture and gram stain as well as clinical presentation which was not consistent with acute meningitis. Cryptococcal antigen (CrAg) testing and fungal culture confirmed 2 cases of cryptococcal meningitis, but only one of the two samples (50.0%) tested positive for *Cryptococcus* on the ME panel, conferring one false negative result (Table 2). Laboratory agreement for viral targets could not be performed on all cases, as not every case had a comparison laboratory test for confirmation. For the positive viral cases, 12/37 (32.4%) had confirmatory CSF testing by IgM/IgG or quantitative PCR. Confirmatory testing was performed in 2/2 (100%) CMV cases, 5/13 (38.5%) HHV-6 cases, 5/7 (71.4%) VZV cases (Table 3). Based on clinico-laboratory review, the overall clinical concordance for viral targets was 98.4%. Clinically discordant viral ME results included HHV-6 (10/13 cases, 76.9%) and CMV (1/2 cases, 50.0%) (Table 2). Further characterization of the HHV-6 positive cases has been previously described (28).

**Table 3:** Positive viral cases by ME panel testing that underwent confirmatory CSF testing.

**Viral Target**	**CSF IgM/IgG**	**Positive CSF confirmatory testing by IgM/IgG**	**CSF Quantitative PCR**	**Positive CSF Quantitative PCR Result**
CMV *n* = 2			2 (100%)	1 (50%)
HHV-6 *n* = 5	3 (60%)		3 (60%)	2 (66%)
VZV *n* = 5	4 (80%)	1 (25%)	3 (60%)	2 (66%)

### Time Analysis and Antimicrobial Usage

Median time from admission to LP was 27.2 h (IQR, 6.2–81.3). In some cases, suspicion for CNS infection occurred days to weeks into an admission. The median time from LP to ME panel results was 1.5 h (IQR, 1.4–1.7).

A total of 416 (59.0%) patients received empiric antimicrobial treatment for CNS infection during their hospital course, of which 104 (25.0%) received acyclovir. Overall median exposure time of acyclovir was 3.1 h (IQR, 0.0–33.1). Twenty-four and forty-eight hours after the ME panel resulted, 28 and 21% of patients remained on acyclovir, respectively. Overall median exposure time of antibiotics was 2.0 h (IQR, 0.0–43.3). Twenty-four and forty-eight hours after the ME panel resulted, 68 (25%) of patients started on empiric therapy remained on antibiotics, respectively. The median time from diagnosis to discontinuation and/or narrowing of antibiotic coverage was 25.6 h (IQR, 3.6–42.5).

Targeted treatment was administered based on ME results in 4 (36.4%) of the clinically discordant cases with average length of treatment of 20 days. The only bacterial false positive, *S. pneumoniae*, received 79 h of targeted antibiotic treatment. Two discordant positive cases for CSF HHV-6 PCR received treatment after the ME panel resulted with duration of exposure of 10 and 45 days. One other discordant positive HHV-6 case also received targeted treatment, though ganciclovir was started before the ME panel resulted due to positive blood HHV-6 PCR. Retrospective review determined chromosomal integration as the cause of HHV-6 positivity in both blood and CSF. ME panel results changed antimicrobial management in four concordant positive cases where targeted antimicrobials were initiated on average 4.9 h (SD 4) after the panel resulted. These included three HHV-6 cases and one HSV-2 case (28).

## Discussion

We are in dire need for rapid and accurate diagnostics for presumed CNS infections given the associated risks of prolonged antimicrobial exposure, current challenges with diagnosis, prolonged length of hospital stays, costs, and the significant morbidity and mortality of undiagnosed and untreated CNS infections. There have been recent major advances in novel diagnostic platforms for CNS infections, including multiplex PCR assays such as the ME panel, 16s ribosomal DNA sequencing, and metagenomic deep sequencing (29). The appeal of these techniques is the ability to evaluate multiple pathogens at one time (30). Evidence from case reports and case series exemplify the potential benefit of these techniques for identifying treatable pathogens (31–38). These exciting aspects are countered by the high costs of novel diagnostic platforms, the local resources required for testing including technical expertise and bioinformatics support for deep sequencing techniques, and the challenge of interpreting results, including the potential for false positive and negative results (30). There remains significant concern with respect to how to use these tests effectively in clinical practice, and how they can aide in the clinical decision-making process. Not only is it essential that we evaluate the performance of such tests against gold standard testing, but understand mechanisms to use these diagnostic approaches to optimize patient care, antimicrobial stewardship, hospital related costs, and patient outcome.

With routine availability of the ME panel at our institution, we found a significant overutilization of the test, as there was minimal or no suspicion for CNS infection in over one-third of ME panel tests ordered. Some institutions have adopted criteria to approve ordering of the ME panel based on clinical assessment and/or CSF profile, while others require approval by infectious disease specialists prior to ordering on any patient (39). Though, if one of the major benefits to the test is in its rapidity of results and negative predictive value, then one must consider the additional time and resources needed to implement these criteria. Another major caveat is that patients at highest risk of CNS infections, including those who are immunocompromised and pediatric patients, frequently present with nonspecific symptoms and atypical CSF profiles. Additionally, CSF can be normal in patients with viral meningoencephalitis, most commonly caused by enteroviral infections in the pediatric population (40–42). In one study of pediatric patients where routine testing was available, the use of the ME panel led to significant reduction of antibiotics in infants between 2 and 12 weeks (41). Though, similar to our results, the authors found that the test was overutilized when routinely available for all patients under the age of 19 years. The authors postulated that one should consider restricting the availability of testing without prior consultation with the pediatric infection team, particularly for patients 1–12 weeks of age presenting with fever to the ER (43).

The potential for discordant results on the ME panel presents another issue. False negative cases have been reported for viral and bacterial pathogens, including *S. agalactiae, S*. *pneumoniae*, HSV, CMV, VZV, HHV-6, and *Cryptococcus* (15, 16, 44–46). There is also relatively sparse data regarding accuracy of positive results for rare bacterial pathogens including *N. meningitidis, L. monocytogenes, and H. influenzae* (44). The test should not be performed in patients suspected of having nosocomial or ventricular drain/device-related infections, as commonly-associated pathogens such as *Staphylococcus* species, *Pseudomonas aeruginosa*, enteric Gram-negative rods (except *E. coli* K1), and *Cutibacterium* (*Propionibacterium) acnes* are not included on the ME panel (4). Additionally, the ME panel detects some pathogens that are not commonly seen in immunocompetent patients, including CMV, HHV-6, VZV, and *Cryptococcus*. Positive results in cases where the pre-test probability is low presents a diagnostic and treatment dilemma, exemplified in particular by the number of clinically insignificant HHV-6 positive cases reported in our previous case series (28). Of note, the patients included in that study were from a broader date range, which accounts for the difference in patient numbers between the two studies. In contrast, false negative results may lead to the inappropriate discontinuation of antimicrobials or not starting antimicrobials when a CNS infection is present (47–50).

A case report also highlights the challenges of relying on the ME panel alone, as one case of tuberculous meningitis (TBM) was misdiagnosed as HSV-1 encephalitis due to a positive HSV-1 PCR result on ME testing, delaying TBM treatment (51). We were not able to analyze the diagnostic testing practices of patients for whom the ME panel was ordered given our retrospective cohort, though further studies are needed to evaluate whether clinicians are overly reliant on the ME panel alone to evaluate patients with possible CNS infections. The ME panel should be interpreted alongside the clinical context, including history and examination, ancillary laboratory testing, and neuroimaging findings (44). Given the overutilization seen in our implementation data, ME panel ordering restrictions should be considered to limit overuse. Further studies are planned to evaluate the impact of ordering restrictions on overall test use and patient outcomes.

There are important limitations of this study including its retrospective design at a single hospital setting and lack of control group. While patient data associated with ME panel results were reviewed independently by multiple physician experts and laboratory and clinical criteria were standardized, these assessments may also have been subject to misclassification. Additionally, this study examined the result time of the ME panel from when the panel was originally introduced and available for ordering at our institution in August of 2016. Time to result was defined as the time the ME panel reaches the laboratory to when it results in the EMR. In some cases early in implementation of the ME panel at our institution, the ME panel was received in the laboratory but not immediately ran on the BioFire^©^ multiplex panel, resulting in longer turnaround times. Longer turnaround times when the panel was first introduced could be explained by lab technicians' unfamiliarity with the testing procedures for the new panel. Although we evaluated time-to-diagnosis and de-escalation of antimicrobial medication, many factors contributed to changes in antimicrobial usage, and we therefore cannot make conclusions on the impact of the ME panel on antimicrobial stewardship. Furthermore, we did not compare antimicrobial usage to the pre-ME panel era. Some values related to time are reported as 0 h, which are marked as the lower limits seen in some data, like culture or test result time. This is due to a pre-determined time of result indicated by our microbiology laboratory. An exposure time of 0 h was indicated in patients for whom antimicrobials were ordered as a one-time-only dose. Finally, due to low incidence rate of CNS infections overall, only a small minority of patients tested positive for any pathogen by the ME panel, limiting our analysis of the significance. Thus, additional studies are required to further assess significance of positive pathogen results. Our study has several strengths as we evaluated a large and diverse patient population, including immunocompromised, transplant, pediatric, and adult patients. Our evaluation of the ME panel performance was based on thorough review of patient clinical data: all participants' relevant clinical notes, radiographic, and laboratory data were reviewed independently of ME results, allowing assessment of assay performance.

In summary, our study demonstrates that routine availability of the ME panel for testing leads to overutilization and potential overreliance of the test for diagnostic purposes. Further studies are needed to gain insight into the role of the ME panel in the diagnostic evaluation and antimicrobial treatment of CNS infections.

## Data Availability

The datasets generated for this study are available on request to the corresponding author.

## Ethics Statement

The Columbia University Irving Medical Center (CUIMC) Institutional Review Board (IRB) approved this study.

## Author Contributions

SarR: data collection and drafting and revising the manuscript. SavR: data collection and drafting the manuscript. PC: data analysis and drafting the manuscript. AB: data collection. NH: data analysis. JZ, BM, SW, WG, MP, and DG: revising the manuscript for intellectual content. DS: data analysis and drafting the manuscript for intellectual content. KT: data collection, drafting and revising the manuscript for intellectual content.

### Conflict of Interest Statement

JZ is supported by the training grant “Training in Pediatric Infectious Diseases” (National Institute of Allergy and Infectious Diseases T32AI007531). DG received research funding from BioFire and has also served on a BioFire scientific advisory panel; this study was not funded by BioFire; DG is also a scientific consultant for Vibrant Sciences. KT is an external consultant, World Health Organization; National Institute of Health “(NIH) Funding Support (K23 NS105935).” The remaining authors declare that the research was conducted in the absence of any commercial or financial relationships that could be construed as a potential conflict of interest.
